# Reliability and acceptability of six station multiple mini-interviews: past-behavioural versus situational questions in postgraduate medical admission

**DOI:** 10.1186/s12909-017-0898-z

**Published:** 2017-03-16

**Authors:** Toru Yamada, Juichi Sato, Hiroshi Yoshimura, Tomoya Okubo, Eiji Hiraoka, Takashi Shiga, Tadao Kubota, Shigeki Fujitani, Junji Machi, Nobutaro Ban

**Affiliations:** 1 Department of General Medicine/Family & Community Medicine, Nagoya University Graduate School of Medicine, Nagoya, Aichi Japan; 2 Department of Internal Medicine, Tokyo Bay Urayasu Ichikawa Medical Center, Urayasu, Chiba Japan; 3 Educational Committee, Prefectural Okinawa Nanbu and Children’s Medical Centre, Haebaru Town, Okinawa Japan; 4 Research Division, The National Center for University Entrance Examinations, Tokyo, Japan; 5 Department of Emergency and Critical Care Medicine, Tokyo Bay Urayasu Ichikawa Medical Center, Urayasu, Chiba Japan; 6 Department of Surgery, Tokyo Bay Urayasu Ichikawa Medical Center, Urayasu, Chiba Japan; 7 Educational Committee, Tokyo Bay Urayasu Ichikawa Medical Center, Urayasu, Chiba Japan; 8 Emergency Medicine and Critical Care Medicine, St. Marianna University, Kawasaki, Kanagawa Japan; 9 Department of Surgery, University of Hawaii, Honolulu, HI USA

**Keywords:** Generalisability theory, Multiple mini-interview, Past behavioural question, Situational question, Reliability, Acceptability, Postgraduate medical admissions

## Abstract

**Background:**

The multiple mini-interview (MMI) is increasingly used for postgraduate medical admissions and in undergraduate settings. MMIs use mostly Situational Questions (SQs) rather than Past-Behavioural Questions (PBQs). A previous study of MMIs in this setting, where PBQs and SQs were asked in the same order, reported that the reliability of PBQs was non-inferior to SQs and that SQs were more acceptable to candidates. The order in which the questions are asked may affect reliability and acceptability of an MMI. This study investigated the reliability of an MMI using both PBQs and SQs, minimising question order bias. Acceptability of PBQs and SQs was also assessed.

**Methods:**

Forty candidates applying for a postgraduate medical admission for 2016–2017 were included; 24 examiners were used. The MMI consisted of six stations with one examiner per station; a PBQ and a SQ were asked at every station, and the order of questions was alternated between stations. Reliability was analysed for scores obtained for PBQs or SQs separately, and for both questions. A post-MMI survey was used to assess the acceptability of PBQs and SQs.

**Results:**

The generalisability (G) coefficients for PBQs only, SQs only, and both questions were 0.87, 0.96, and 0.80, respectively. Decision studies suggested that a four-station MMI would also be sufficiently reliable (G-coefficients 0.82 and 0.94 for PBQs and SQs, respectively). In total, 83% of participants were satisfied with the MMI. In terms of face validity, PBQs were more acceptable than SQs for candidates (*p* = 0.01), but equally acceptable for examiners (88% vs. 83% positive responses for PBQs vs. SQs; *p* = 0.377). Candidates preferred PBQs to SQs when asked to choose one, though this difference was not significant (*p* = 0.081); examiners showed a clear preference for PBQs (*p* = 0.007).

**Conclusions:**

Reliability and acceptability of six-station MMI were good among 40 postgraduate candidates; modelling suggested that four stations would also be reliable. SQs were more reliable than PBQs. Candidates found PBQs more acceptable than SQs and examiners preferred PBQs when they had to choose between the two. Our findings suggest that it is better to ask both PBQs and SQs during an MMI to maximise acceptability.

**Electronic supplementary material:**

The online version of this article (doi:10.1186/s12909-017-0898-z) contains supplementary material, which is available to authorized users.

## Background

The single-station personal interview (SSPI) is widely used for medical and non-medical admission interviews. However, the SSPI has two significant problems: context specificity [1, 2] and interviewer bias (i.e., the halo, or ‘similar-to-me’ effect) [2]. The multiple mini-interview (MMI), first used in 2004, is an interview method designed to overcome these problems [2].

MMI is increasingly acknowledged as an alternative method for under- or postgraduate medical admissions in the United States [3, 4], the United Kingdom [5, 6], Canada [2, 7–10], and non-Western countries [11]. Reliability, acceptability, and validity are important requirements for an interview method [12]. To ensure reliability, MMI is thought to require seven to twelve stations, with one examiner per station [8, 9, 13], and has been reported to be similar or superior to SSPI in acceptability [2, 6, 9, 14, 15].

SSPIs and MMIs either utilise situational questions (SQs) or past-behavioural questions (PBQs). SQs ask candidates what they would do in a certain hypothetical situation, whereas PBQs ask about the candidate’s actual experience. Until recently, it was common to ask SQs rather than PBQs in MMIs [16, 17], although both PBQs and SQs have been widely used in SSPIs [18]. Studies of non-medical admissions have demonstrated that reliability and acceptability are similar for PBQs and SQs in SSPIs, though PBQs have a higher predictive validity for high-complexity jobs, compared with SQs [16, 18]. One study [17] reported that the reliability of PBQs was non-inferior to SQs for an MMI-format postgraduate medical admission interview and that an MMI with five stations and two examiners per station was sufficient to ensure reliability when a structured approach was used. However, the study generated several additional questions about MMIs that need further investigation. Candidates were asked two questions per station: a PBQ and an SQ, always in that order. The answer to the first question may have affected the answer to the second, as they were asked at the same station; the reliability of SQs and the acceptability of PBQs and SQs may therefore have been affected by the fixed order of questions. Candidates in the study considered SQs more acceptable and easier to answer than PBQs, which may have been because they had adapted to the interview and were feeling more comfortable when answering the second question (an SQ). An investigation of the reliability of MMI using both types of questions, in different orders, would be of value.

This study aimed to investigate the reliability of PBQs, SQs, and both question types together using a six-station MMI with one examiner per station and an alternating question order at each station to minimise question order bias. It also aimed to assess the acceptability of PBQs and SQs among candidates and examiners.

## Methods

### Settings and participants

After completing medical school, graduates in Japan obtain their medical licence by passing a national board examination. This is followed by the completion of the two-year National Obligatory Initial Postgraduate Clinical Training Programme (NOIPCTP) [17, 19], after which physicians hold unlimited licenses and must obtain specialty training to become board-certified specialists. This study was conducted among individuals applying for specialty training in internal medicine, surgery, and emergency medicine. The selection was held on two days in September and October 2015 and two days in September 2016 at Tokyo Bay Urayasu Ichikawa Medical Center (TBUIMC), a midsize community hospital in Chiba, Japan, which has used MMIs since 2013 [17]. There were 24 examiners (23 men and one woman) involved over the 4 days, all of whom were licensed attending physicians in internal medicine, surgery, or emergency medicine at TBUIMC. All candidates, regardless of the specialty for which they were applying or their post-graduate year level, were examined by all examiners in attendance on each day. Examiners were randomly allocated to stations and stayed at the same station throughout the process.

### Intervention

This study used six stations, each with one examiner assigned. There were two reasons for the reduction in the number of stations from the usual ten to six. First, a previous study in this setting demonstrated that an MMI with six stations and one examiner per station could ensure good reliability [17]. Second was the issue of cost. In Japan, especially in small to midsize community hospitals, attending physicians as examiners are a very limited resource. Numbers of examiners were therefore reduced as much as possible while maintaining reliability.

In 1999, the Accreditation Council for Graduate Medical Education introduced six domains of clinical competency for physicians: medical knowledge; patient care and procedural skills (PCPS); system-based practice (SBP); interpersonal and communication skills (ICS); practice-based learning and improvement (PBLI); and professionalism (Pro) [20, 21]. Each domain included two to eight sub-domains [20]. Each station was set up to examine one of the domains of competence, with one station for each of PCPS, PBLI, ICS, and SBP, and two stations for Pro. The domain of medical knowledge was excluded because it was not considered appropriate for assessment through MMI. Two stations were set up for Pro because the TBUIMC training programme committee regarded it the most important of the six domains. Each domain was randomly allocated two of its associated sub-domains (one per question) for each station (Table 1). All of the PBQs and SQs were constructed based on questions previously used in MMIs at TBUIMC, some of which have been previously reported [17].

**Table 1:** Competencies (domains), sub-domains and question types for the multiple mini-interview stations

Station	Competency	Sub-domain	Question format
Station 1	PCPS (IV.A.5.a)^a^	(1)^b^ Managing patient problems (treatment, health promotion)	PBQ
		(2)^b^ Performing procedures competently	SQ
Station 2	PBLI (IV.A.5.c)^a^	(7)^b^ Using information technology to optimize learning	PBQ
		(8)^b^ Participating in the education of others	SQ
Station 3	ICS (IV.A.5.d)^a^	(1)^b^ Communicating effectively with patients	PBQ
		(3)^b^ Working effectively as a member or leader of a team	SQ
Station 4	Pro1 (IV.A.5.e)^a^	(4)^b^ Maintaining accountability to patients, society and the profession	PBQ
		(3)^b^ Having respect for patient privacy and autonomy	SQ
Station 5	Pro2 (IV.A.5.e)^a^	(2)^b^ Showing responsiveness to patient needs that supersedes self-interest	PBQ
		(5)^b^ Showing sensitivity and responsiveness to a diverse patient population	SQ
Station 6	SBP (IV.A.5.f)^a^	(6)^b^ Participating in identifying system errors and implementing potential systems solutions	PBQ
		(2)^b^ Coordinating patient care within the health care system	SQ

^a^Competency number in the Accreditation Council for Graduate Medical Education (ACGME) common programme requirements [21]

^b^Sub-domain number within the competency in the ACGME common programme requirements [21]

*PCPS* patient care and procedural skills, *PBLI* practice-based learning and improvement, *ICS* interpersonal and communication skills, *Pro* professionalism, *SBP* system-based practice, *PBQ* past behaviour question, *SQ* situational question

One PBQ and one SQ were asked to every candidate at every station. The six stations were divided into two groups of three stations each: in the first group, the PBQ was asked first; and in the second group, the SQ was asked first. Candidates were assessed at group one and group two stations in alternate order to minimise question order bias. Each station was allotted 10 min, with 5 min allowed for each question and a 1-min break between stations.

Before asking a PBQ, the candidate was informed that the question was about their experience during their junior residency; the Situation-Task-Action-Result (STAR) approach was applied to guide the answers [17, 22]. Before asking an SQ, the examiner explained that the question was about what would happen if they were to work as a senior resident at TBUIMC; a hypothetical scenario was described: candidates were presented with an ethical dilemma and asked what they would do, selecting one of two or more mutually exclusive possible courses of action [17, 18]. This was followed by structured probing by the examiner [16, 17].

All candidates were fully informed about the logistics of the MMI by email in advance and orally on the day of the MMI; all agreed for the results to be published. No information about which competency sub-domains would be assessed was provided to the candidates. Sixteen (67%) of the 24 examiners had previous experience in MMIs at TBUIMC and had therefore undergone training in the previous year. The remaining eight (33%) first-time examiners were trained prior to beginning the MMI using a method previously described [17]. Changes made to earlier methods were detailed. Examiners were given general instructions to keep the interview questions on track and to minimise close rapport-building with the candidates during the examination.

To assess candidates, examiners used rating rubrics that have been used for interviews at TBUIMC since 2013 [17] (Additional file 1). These included evaluation of three areas: ‘communication skills’, ‘strength and certainty of the answer’, and ‘suitability for the programme’. A five-point scale, each point defined with a descriptor, was used to score each area. These three rubrics were used per question. On the day of the MMI, a group of candidates rotated through all six stations.

### Post-MMI survey (Table 2)

**Table 2:** Post-multiple mini-interview survey (*n* = 64: candidates 40, examiners 24)

Question		Number of PRs	Mean score	*P*-value
1(C)	In general, the current MMI allowed me to express my own abilities accurately (general satisfaction).	33/40 (83%)	PR 3.152 NR 2.000	<0.001
1(E)	In general, the current MMI allowed me to assess the candidates’ abilities accurately (general satisfaction).	20/24 (83%)	PR 3.200 NR 2.000	<0.001
2(C)	Questions on “experience during junior residency” allowed me to express my abilities accurately.	38/40 (95%)	3.23	0.010
	Questions on “if you work as a senior resident at TBUIMC” allowed me to express my abilities accurately.	30/40 (75%)	2.950	
2(E)	PBQs allowed me to assess candidates’ abilities accurately.	21/24 (88%)	3.13	0.38
	SQs allowed me to assess candidates’ abilities accurately.	20/24 (83%)	3.000	
3(C)	I had sufficient time to present my ideas for questions on “experience during junior residency.”	33/40 (83%)	3.050	1.000
	I had sufficient time to present my ideas for questions on “if you work as a senior resident at this hospital.”	34/40 (85%)	3.050	
3(E)	For the PBQs, I had sufficient time to manage the sessions.	23/24 (96%)	3.500	0.42
	For the SQs, I had sufficient time to manage sessions.	22/24 (92%)	3.46	
4(C)	I did not have any difficulties answering questions on “experience during junior residency.”	33/40 (83%)	3.08	0.08
	I did not have any difficulties answering questions on “if you work as a senior resident at this hospital.”	27/40 (68%)	2.800	
4(E)	I did not have any difficulties asking the PBQs.	19/24 (79%)	3.33	0.15
	I did not have any difficulties asking the SQs.	17/24 (71%)	3.04	
5(C)	The current MMI is fairer than the SSPI.	37/40 (93%)	PR 3.541 NR 1.667	<0.001
5(E)	The current MMI is fairer than the SSPI.	23/24 (96%)	PR 3.435 NR 2.000	<0.001
6(C)	The workload of the current MMI is acceptable.	37/40 (93%)	PR 3.514 NR 1.667	<0.001
6(E)	The workload of the current MMI is acceptable.	23/24 (96%)	PR 3.478 NR 2.000	<0.001
		Proportion of answers (%)		
7(C)	Would you choose either one of the two question formats “experience during junior residency” and “if you work as a senior resident at this hospital” or both, to express your abilities?	Both questions One question	34/40 (85%) 6/40 (15%)	<0.001
	Please write the reason in the space provided for free comments.			
7(E)	Would you choose either one of the two question formats, PBQ and SQ, or both to assess candidates’ abilities?	Both questions One question	20/24 (83%) 4/24 (17%)	0
	Please write the reason in the space provided for free comments.			
8(C)	If you had to select only one type of question, which would you want to answer to express your abilities better—“experience during junior residency” or “if you work as a senior resident in this hospital?”	PBQ SQ	26/40 (65%) 14/40 (35%)	0.08
	Please write the reason in the space provided for free comments.			
8(E)	If you had to select only one type of question, which would you want to ask to assess candidates’ abilities, PBQs or SQs?	PBQ SQ	19/24 (79%) 5/24 (21%)	0.01
	Please write the reason in the space provided for free comments.			

(C): Questions for candidates

(E): Questions for examiners

*PR* Positive response includes “mostly agree” and “agree”

*NR* Negative response includes “mostly disagree” and “disagree”

*MMI* multiple mini-interview

*PBQ* past behavioural question

*SQ* situational question

*SSPI* single station personal interview

At the end of the MMI process, all candidates and examiners answered a brief, anonymous survey, which was based on post-MMI surveys used at TBUIMC since 2013 [17]. In general, overall acceptability of MMI is evaluated by integration of face validity, candidate (or examiner) reaction, fairness, and feasibility. Therefore, to assess face validity, participants were asked about general satisfaction with the MMI method (Table 2: 1C, 1E), candidates’ satisfaction with the abilities assessed, and examiners’ opinions about the accuracy of assessing these abilities based on PBQ and SQ formats (Table 2: 2C, 2E); to assess candidate or examiner reaction, they were asked about the adequacy of time and ease in answering or asking questions in both formats (Table 2: 3C, 3E, 4C, 4E); and to assess general fairness, comparisons were made with SSPIs and questions asked about the acceptability of workloads (Table 2: 5C, 5E, 6C, 6E). All responses were recorded using a four-point Likert scale (disagree [1], mostly disagree [2], mostly agree [3], or agree [4]). Participants were also asked two additional questions: which they preferred, inclusion in the interview of both question formats, or only one; and, if they had to select only one type, which of PBQs or SQs would they choose. Space was provided for comments about these two questions. Participants were informed that individual survey answers would be kept confidential, used for research purposes, and not affect selection decisions.

### Data analysis

To determine reliability, the MMI scores were analysed using generalisability (G) theory. We used Mplus v5.21 (Muthén & Muthén, Los Angeles, CA, USA) for G and decision (D) studies. The model was adjusted for the candidate’s ability, rubrics, the station, and residual variance. As each station involved both a PBQ and SQ, three patterns of variance components were modelled: only PBQ, only SQ, and both PBQ and SQ. For example, candidate’s ability, rubrics, station PBQs, station SQs, and residual variance were set as variance components in analysing the results of PBQs and SQs. For the analysis of the post-MMI surveys, R v3.1.3 (R Foundation for Statistical Computing, Vienna, Austria) was used for paired t-tests, one-sample t-tests and binominal tests. Paired t-tests were used to compare the effectiveness of PBQs and SQs in expressing or assessing candidates’ abilities, time management, and ease of questioning/answering. For general satisfaction, fairness, and workload, combined scores of ‘agree’ and ‘mostly agree’ categories were compared with combined ‘mostly disagree’ and ‘disagree’ categories using a one-sample t-test. A binominal test was used to compare participants’ preferences for the inclusion of single or dual question formats in one interview and for PBQs or SQs.

## Results

### Participants

A total of 40 candidates applied and all went through the MMI. The mean age was 28.1 (range 25–48) years and 31 (77.5%) were men. The mean scores for PBQs and SQs were 4.00 (standard deviation [SD] 0.91) and 4.00 (SD 0.90), respectively.

### Reliability

We calculated the G-coefficients used in G and D studies. The estimated variance components of candidates’ ability on PBQs, SQs, and both questions were 0.312–0.476 (Table 3), suggesting that the candidates were not a standardised group, but had moderate differences. The estimated variance components of the stations were small, suggesting that the level of difficulty in each station was adequate. In the D study, the G-coefficients for PBQs alone, SQs alone, and both question formats were 0.87, 0.96, and 0.80, respectively, with six stations and one examiner (Table 4). These values were 0.82, 0.94, and 0.73, respectively, when this was reduced to four stations.

**Table 3:** Estimated variance components for each variable included in the model, stratified by question format (PBQs, SQs, or PBQs and SQs) (*n* = 40)

	Variance components		
	PBQs and SQs	PBQs	SQs
Factor: Candidate’s ability	0.361	0.312	0.476
Factor: Rubrics	0.004	0.000	0.000
Factor:			
Station PBQs	0.241	0.255	
Station SQs	0.243		0.080
Residual variance	0.266	0.299	0.532

*PBQ* past behavioural question, *SQ* situational question

**Table 4:** Results of the decision study, showing G-coefficients for MMIs with four to eight stations, stratified by question format (PBQs, SQs, or PBQs and SQs) (*n* = 40)

Number of stations^a^	G-coefficient for PBQs and SQs	G-coefficient for PBQs	G-coefficient for SQs
4	0.73	0.82	0.94
5	0.77	0.85	0.95
6	0.80	0.87	0.96
7	0.82	0.89	0.97
8	0.84	0.90	0.97

Three rating rubrics per question and one examiner per station

*G-coefficient* generalisability coefficient, *MMI* multiple mini-interview, *PBQ* past behavioural question, *SQ* situational question

### Acceptability

All 64 participants (*n* = 40 candidates and *n* = 24 examiners) answered the post-MMI survey regarding acceptability. Overall, 53/64 (83%) participants were satisfied with the MMI in this study (Table 2). In terms of face validity, PBQs were more acceptable than SQs for candidates (positive responses [PR] in 38/40 [95%] vs. 30/40 [75%] for PBQs vs. SQs; *p* = 0.01; Table 2), but equally acceptable for examiners (PR in 21/24 [88%] vs. 20/24 [83%] for PBQs vs. SQs; *p* = 0.377). More candidates felt that the PBQs were easy to answer than did the SQs, but the difference was not statistically significant (PR in 33/40 [83%] vs. 27/40 [68%] for PBQs vs. SQs; *p* = 0.078). Of the 40 candidates and 24 examiners, 37 (93%) and 23 (96%), respectively, reported both that the MMI format was fairer than the SSPI and that the workload was acceptable; 34/40 (85%) candidates and 20/24 (83%) examiners preferred to use both question formats rather than only one; and more candidates and examiners chose PBQs over SQs when asked to select only one, though only in the latter group was this difference statistically significant (candidate PR 26/40 [65%] vs. 14/40 [35%] and examiner PR 19/24 [79%] vs. 5/24 [21%] for PBQs vs. SQs; *p* = 0.081 and *p* = 0.007, respectively).

## Discussion

We conducted an MMI with six stations and one examiner per station and found that the overall performance of this MMI format was reliable. In contrast to previous work in this setting, the reliability of SQs was superior to PBQs, which may be the result of minimising question order bias. As previously described, PBQs have been shown to have good reliability and validity in non-medical admissions, particularly showing a higher predictive validity for high-complexity jobs when compared with SQs [16, 18]. A Canadian study also reported that PBQs were more reliable than SQs in medical admissions [23]. We therefore tried to compare the reliability of PBQs with SQs in the setting of postgraduate medical admission in Japan because applicants are likely to have had more experience and more exposure to complex work than undergraduates. Our study showed that the reliability of SQs was better than PBQs. However, in general, G-coefficient scores of 0.80 or higher are considered to represent excellent reliability. Therefore, both PBQs and SQs were sufficiently reliable for junior residents under NOIPCTP in Japan. Reliability of both PBQs and SQs were better than in a previous study in this setting [17]. Other than minimising question order bias, the good reliability observed may be because two-thirds of the examiners had previous experience in MMIs at TBUIMC and the remainder were trained in advance [17]. The examiners were therefore sufficiently similar and the assessments of each examiner had a certain amount of homogeneity.

This study also showed that an MMI with four stations and one examiner per station using PBQs or SQs was sufficiently reliable, suggesting that MMIs can be conducted with fewer examiners and stations if context specificity, interviewer bias, and training of examiners are carefully accounted for. This finding may contribute to improvements in MMIs for postgraduate medical admissions. However, acceptability may decrease if MMIs use either PBQs only or SQs only, as over 80% of participants preferred to use both question formats rather than only one. Reliability of SQs was very high, but this may have been because SQs evaluated a narrower range of candidates’ abilities, suggesting that the validity of an MMI using SQs alone may not be satisfactory. We plan to evaluate the validity of an SQ-only MMI method in the future. In addition, reliability was analysed in the context of two questions per station. Future studies should investigate the reliability of each type of question when asked alone at one station if we want to determine the reliability of PBQs only or SQs only with more accuracy.

Overall, over 80% of participants gave positive responses (‘mostly agree’ or ‘agree’) to most questions in the post-MMI survey; 83% of participants were satisfied with the MMI method used and over 93% were satisfied in terms of fairness and workload, suggesting that the overall acceptability of this MMI method was good. In particular, the acceptance of the workload by 96% of examiners suggests that this MMI method may be feasible for use in midsize community hospitals like TBUIMC. In contrast to previous findings among candidates at TBUIMC, PBQs were more acceptable and easier to answer than SQs in this study [17]. Minimising question order bias may provide a more accurate estimate of acceptability of the MMI. The majority of participants indicated that both questions were acceptable but examiners clearly preferred PBQs when they were asked to choose between them (*p* = 0.007). Based on the free comments in the surveys, some of the candidates and examiners who preferred PBQs to SQs felt that PBQs assessed candidates’ actual experience and therefore seemed more reliable; those who preferred SQs to PBQs felt that SQs used a complicated scenario with an ethical dilemma and therefore seemed more suitable for evaluating a candidate’s ability. Irrespective of these differences, 85% of candidates and 83% of examiners preferred to use both PBQs and SQs, instead of only one question format. The most frequently listed reason for this was that using both question formats provided more chances to express or evaluate abilities. With these findings in mind, we suggest it would be preferable to use both question formats to maximise acceptability of the MMI. However, reliability and acceptability are only two aspects of question format; validity is also important aspect and requires consideration and further investigation.

This study had limitations. First, it was conducted in one medical centre, which does not allow for generalisation to other medical programmes. Therefore, multi-centre studies are needed to further investigate the reproducibility of these findings. Second, it is usual, in MMIs, for each ability to be assessed by a separate examiner at each station. In this study, a single examiner asked both a PBQ and a SQ at each station. This was potentially a major source of bias. However, we arranged for the conditions of the two types of question to be the same and therefore thought it would not be a problem when comparing PBQs with SQs.

## Conclusions

This MMI method, with six stations, one examiner per station, and PBQ and SQ question formats that alternated in order at each station, showed good reliability and acceptability. SQs were more reliable than PBQs. Modelling suggested that an MMI with four stations and one examiner per station using either question format may be sufficiently reliable. Candidates found PBQs more acceptable than SQs and examiners preferred PBQs when they had to choose between the two. Our findings suggest that it is better to ask both PBQs and SQs during an MMI to maximise the acceptability of the assessment.

## Additional file

Additional file 1: Rating rubrics. (XLSX 11 kb)
